# Avidity maturation of anti‐spike IgG after vaccination in COVID‐19 convalescent vs COVID‐19 naïve patients

**DOI:** 10.1111/apm.13489

**Published:** 2024-11-07

**Authors:** Emma Löfström, Anna Eringfält, Arne Kötz, Johan Tham, Johan Undén

**Affiliations:** ^1^ Department of Clinical Sciences Lund University, Office DCSL Lund Sweden; ^2^ Department of Clinical Microbiology Hallands hospital Halmstad Halmstad Sweden; ^3^ Department of Translational Medicine, Clinical Infection Medicine Lund University, Skåne University Hospital Malmö Sweden; ^4^ Department of Operation and Intensive care Hallands hospital Halmstad Halmstad Sweden

**Keywords:** Avidity index, anti‐spike IgG, COVID‐19, immune response, vaccination

## Abstract

Antibodies and avidity maturation contribute to long‐lasting immunity, and previous COVID‐19 seems to enhance the immune response after vaccination. The aim of this study was to compare the immune response after vaccination between COVID‐19 convalescents and naïve patients. Blood samples from COVID‐19 convalescents and naïve patients, taken 1, 3 and 6 months after the second dose of vaccine (mRNA‐vaccine BNT162b2), were analysed for anti‐spike IgG and avidity. Questionnaires concerning side effects were used. Thirty‐one patients in the COVID‐19 cohort and 30 patients in the naïve cohort were included. High levels of anti‐spike IgG and avidity index were seen. Anti‐spike IgG were significantly higher in the COVID‐19 cohort and declining (median 1250, 566, 282 RU/ml vs 565, 187, 65 RU/ml). Avidity did not change over time (median at 6 months 78% vs 65%). The most common side effects were pain at the injection site, malaise and headache. In conclusion, high levels of anti‐spike IgG after vaccination were seen and most patients developed high‐avidity antibodies, although antibody levels and avidity were higher in the COVID‐19 cohort. Over time, the levels of anti‐spike IgG declined, yet avidity remained high. Side effects did not differ between groups and were of short duration.

The pandemic era of COVID‐19 heavily affected people all over the world. Initially, little was known about the new virus, SARS‐CoV‐2, and how the pandemic would develop. Now, in the post‐pandemic era, it is clear that the development of vaccine against SARS‐CoV‐2 saved millions of lives and contributed to the end of the pandemic [1, 2]. However, COVID‐19 still has a large impact on healthcare systems. For elderly and fragile patients, or individuals with immune suppression, COVID‐19 can still cause severe illness [3, 4]. Furthermore, new variants of concern might continue to emerge. Therefore, understanding how long‐lasting and protective immunity against SARS‐CoV‐2 develops and is maintained is crucial.

The mRNA‐vaccine BNT 162b2 (Comirnaty, Pfizer‐BioNTech) was one of the first vaccine candidates that was used clinically. A phase II‐study showed that two doses of vaccine, with 21 days between, resulted in high levels of neutralizing antibodies as well as a strong T‐cell response [5]. A safety and efficacy study showed 95% protection against COVID‐19 and only short‐term mild–moderate side effects such as pain at injection site and headache [6]. This vaccination did not only protect vaccinated individuals from severe COVID‐19 but also contributed to the end of the pandemic. However, when vaccinations were initiated, it was not known for how long the immunity would last and how new virus variants would influence the vaccine‐efficacy and hence the pandemic situation, indicating a need for further research of the immune response after vaccination, as well as after natural infection [7]. It is also important to gain knowledge about the side effects of the vaccine to ensure a reasonable risk–benefit balance, especially for extensive vaccinations as during the pandemic [8].

The humoral immune response to vaccines, as well as to natural infection, can be assessed in many ways. To assess the response that leads to protective immunity, the gold standard is to measure the neutralizing capacity of the antibodies using living virus in cell‐cultures, but this is time consuming and requires biosafety level 3 laboratories [9]. An easier method is to measure the levels of antibodies with an ELISA assay. The measured antibody should be directed to a surface epitope in order to presumably have a neutralizing capacity, for example the levels of antibodies against the spike protein of SARS‐CoV‐2. ELISA is an uncomplicated assay and available in most clinical laboratories, and it has been shown that the levels of anti‐spike IgG can be used as a rough estimate of the neutralizing capacity of SARS‐CoV‐2 [10, 11]. Another aspect of the humoral immune response is the avidity of the antibodies, specifically the functional affinity or the overall strength between the antibodies and the antigen. It has been shown that the production of high‐avidity antibodies is essential to develop long‐lasting and protective immunity in several viral infections [12, 13, 14].

The immune response after COVID‐19 differs significantly to the immune response after vaccination. Natural infection gives rise to relatively low levels of anti‐spike IgG and failure of avidity maturation, whilst two doses of vaccine give rise to high levels of anti‐spike IgG and maturation of high‐avidity antibodies [15, 16, 17]. Over time, levels of anti‐spike IgG declines after both infection and vaccination, which is the rational for the need of vaccine boosters [18]. A previous COVID‐19 infection, prior to the vaccination, seems to enhance the immune response with even higher levels of antibodies, neutralizing titres and avidity index [10, 19]. Further understanding of the impact of infection before vaccination may affect vaccine strategies and vaccine priorities. Also, it is not known if side effects after vaccination differ between these groups.

The aim of the study was to compare the immune response, focusing on anti‐spike IgG levels and avidity index, after the second dose of vaccine between a COVID‐19 convalescent cohort and a COVID‐19 naïve cohort. The second aim was to describe the side effects that were reported after the vaccinations.

## METHODS

### Study participants

Patients were sought from the vaccine substudy (part II) of an ongoing study, ‘COVID‐19 Symptoms and Immunity’, in the county of Halland, Sweden. In this study, patients were prospectively included into two cohorts before vaccination against SARS‐CoV‐2. The first cohort (*n* = 132) was already participating in part I of the study and had a PCR‐confirmed COVID‐19 during 2020. The second cohort (*n* = 37) was recruited during January–March 2021 and consisted of healthcare personal with no former COVID‐19. An absence of symptoms of previous or ongoing COVID‐19 and a negative test for nucleocapsid‐antibodies against SARS‐CoV‐2 was required for inclusion into the second cohort. In both cohorts, patients were followed after vaccination against SARS‐CoV‐2 with weekly questionnaires, to register side effects as well as to detect new infections. Blood samples were taken at 1 (±7 days), 3 (±10 days) and 6 months (±10 days) after the second dose of vaccine.

As the majority in the second cohort (33 out of 37) were vaccinated with mRNA BNT 162b2 (Comirnaty (tozinameran), Pfizer‐BioNTech), only patients vaccinated with this vaccine, and with blood samples from at least two time points, were included to this study.

### Questionnaires

Seven days after both the first and the second doses of vaccine, a questionnaire concerning side effects was sent to all participants. A digital system approved for research and healthcare data, Entermedic (Entergate AB), was used and text messages containing a link to the questionnaire was sent. For side effects, the participants could tick from a list with nine common side effects (fever, chills, headache, malaise, fatigue, headache, pain at injection site, swollen/reddened at injection site, allergic reaction) and/or describe in free text. There were also questions concerning the duration of side effects and if these symptoms had caused sick leave.

### Anti‐spike IgG

An ELISA assay, Anti‐SARS‐CoV‐2 QuantiVac ELISA (Euroimmun), was used manually according to the manufacturer's instructions to analyse the levels of anti‐spike IgG. The antigen in the ELISA is the S1 domain of the spike protein of SARS‐CoV‐2, isolate Wuhan‐Hu‐1. Levels ≥11 RU/ml was considered as positive, <11 – ≥8 RU/ml borderline and <8 RU/ml as negative according to manufacturer. When the IgG levels were above the linear range of the assay, the sample was diluted and reanalysed.

### Avidity index

The same ELISA assay was used, but with the addition of an extra step with urea as the chaotropic agent, previously described by Löfström et al, except that 7 M urea was used instead [20]. The concentration of 7 M urea was chosen after initial optimization experiments with samples taken from 4 patients at 1 month after primary SARS‐CoV‐2 infection, 1 month after the second dose of vaccine and 6 months after the second dose of vaccine. If necessary, samples were diluted to fit in the linear range of the assay. Briefly, the samples were analysed in duplicates and urea mixed with washing buffert was added to one well and to the other well pure washing buffert was added instead of urea. After incubation for 10 min, the assay was performed according to the manufacturer instructions, but with the addition of an extra washing cycle. The avidity index was described as OD_urea_/OD_reference_ and multiplied by 100 to be expressed as percentage. In every run, a positive sample was used as a positive control. The coefficient of variation (CV%) for the avidity index was 8%. From the optimization experiments, the definition of low‐avidity antibodies was set to avidity index ≤20% and the definition of high‐avidity antibodies was set to avidity index ≥60%.

### Statistics

All statistics were done in SPSS version 29. For differences over time within the cohorts, Wilcoxon signed ranks test was used, and for differences between the cohorts, Mann–Whitney was used. For differences in reported side effects, chi‐squared test was used. A *p* value of <0.05 was considered significant.

## RESULTS

In the first cohort (COVID‐19 cohort), only 59 patients were vaccinated with mRNA BNT 162b2 (Comirnaty, Pfizer‐BioNTech) and 31 of them met the other criteria with at least two blood samples. In the second cohort (COVID‐19 naïve cohort), 30 patients met the criteria for inclusion, so in total 61 patients formed the basis for this study. For basic characteristics such as age, sex and comorbidities, see Table 1. The mean time between the two doses of vaccine was 44 days in the COVID‐19 cohort and 45 days in the COVID‐19 naïve cohort. The mean time between the PCR‐confirmed COVID‐19 and the first dose of vaccine in the COVID‐19 cohort was 8,5 months (median 10 months, range 2–14 months).

**Table 1:** Basic characteristics

	COVID‐19 cohort (*n* = 31)	COVID‐19 naive cohort (*n* = 30)
Age, median (range)	51 years (26–65)	51 years (24–65)
Time between COVID‐19 and first dose of vaccine, median (range)	10 months (2–14)	–
Sex		
Female	90% (*n* = 28)	73% (*n* = 22)
Male	10% (*n* = 3)	27% (*n* = 8)
Comorbidities	23% (*n* = 7)	37% (*n* = 11)
Hypertension	10% (*n* = 3)	10% (*n* = 3)
Asthma	–	6% (*n* = 2)
Inflammatory Bowel Disease	3% (*n* = 1)	3% (*n* = 1)
Psychatric disorder	3% (*n* = 1)	3% (*n* = 1)
Epilepsi	–	3% (*n* = 1)
Diabetes mellitus type 1	–	3% (*n* = 1)
Migraine	3% (*n* = 1)	3% (*n* = 1)
Angina pectoris	–	3% (*n* = 1)
Irritable Bowel Syndrome	3% (*n* = 1)	–
Immunosuppressive treatment	–	3% (*n* = 1)

**Fig. 1:**
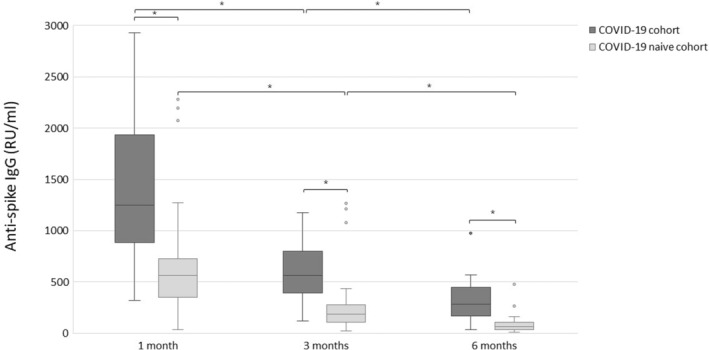
Anti‐spike IgG (RU/ml) after the second dose of vaccine in the COVID‐19 cohort and the COVID‐19 naïve cohort. *p < 0.05.

**Fig. 2:**
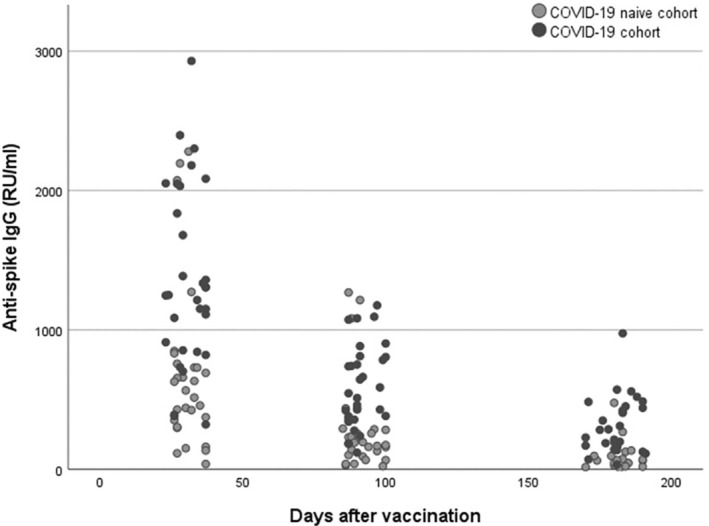
Anti‐spike IgG (RU/ml) after the second dose of vaccine in the COVID‐19 cohort and the COVID‐19 naïve cohort.

**Fig. 3:**
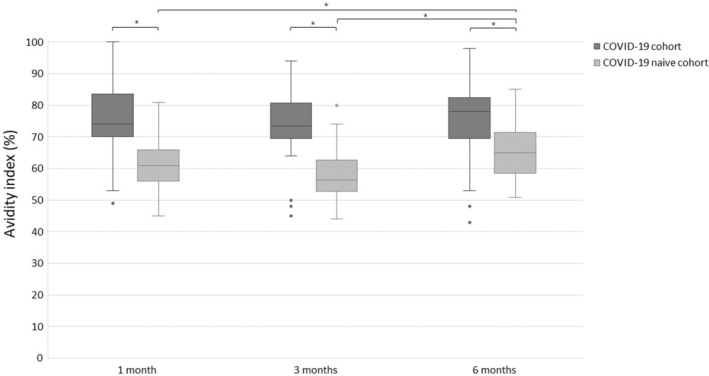
Avidity index (%) of anti‐spike IgG after the second dose of vaccine in the COVID‐19 cohort and the COVID‐19 naïve cohort. *p < 0.05.

**Fig. 4:**
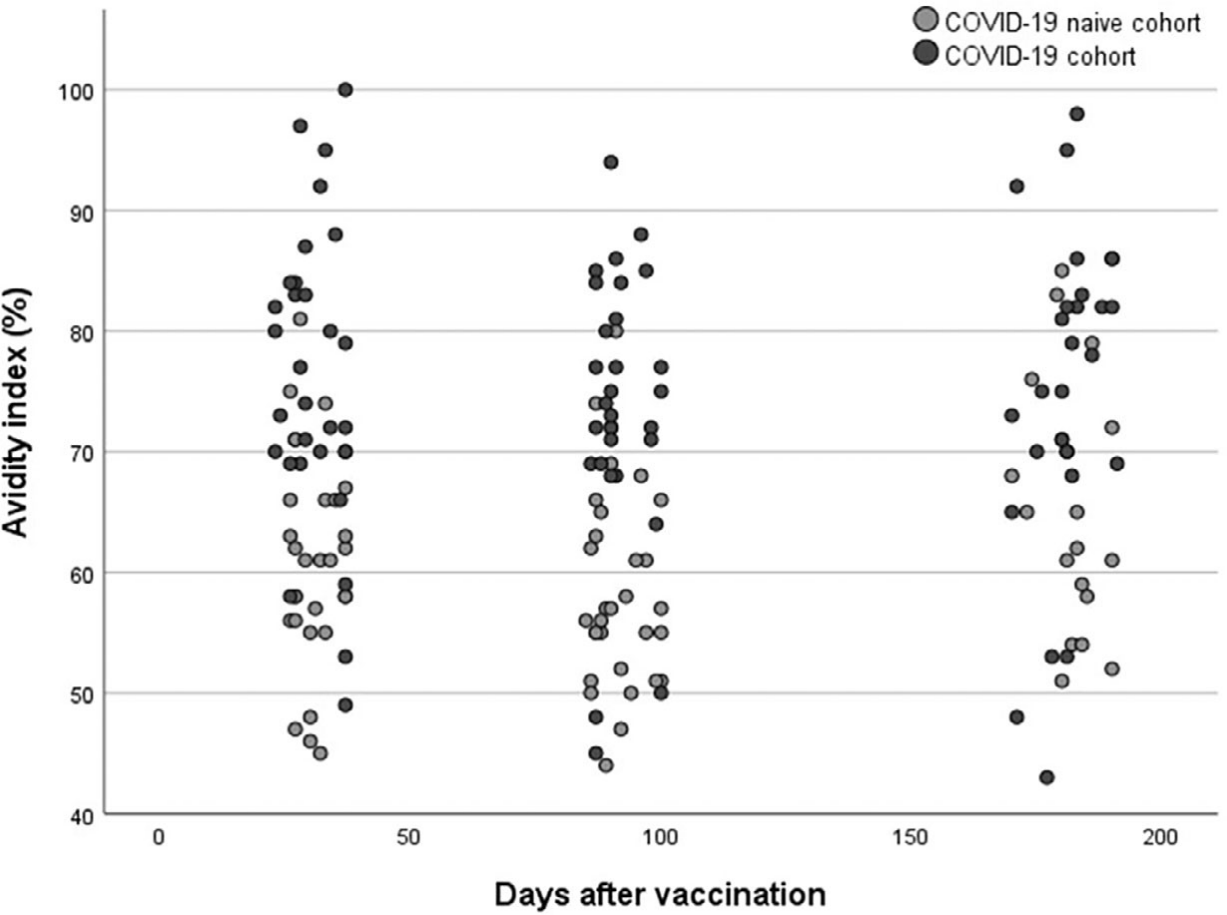
Avidity index (%) of anti‐spike IgG after the second dose of vaccine in the COVID‐19 cohort and the COVID‐19 naïve cohort.

The anti‐spike IgG levels decreased significantly over time in both the COVID‐19 cohort (median value at 1, 3 and 6 months: 1250, 566, 282 RU/ml, *p* < 0,001) and in the COVID‐19 naïve cohort (median value at 1, 3 and 6 months: 565, 187, 65 RU/ml, *p* < 0,001) after the second dose of vaccine, see Figs. 1 and 2. Avidity index increased significantly (*p* < 0.05) in the COVID‐19 naïve cohort from 1 to 6 months and between 3 and 6 months (median value at 1, 3 and 6 months: 61%, 57% and 65%). In the COVID‐19 cohort, there was no statistically significant differences of the avidity index (*p* > 0.05) over time (median value at 1, 3 and 6 months: 74%, 74%, 78%), see Figs. 3 and 4.

Between the cohorts, there was a significant difference (*p* < 0.05) in the levels of anti‐spike IgG and avidity index at all time points with higher levels and higher avidity index in the COVID‐19 cohort, see Figs. 1 and 3. The difference of the median value of the avidity index between the cohorts was at most 17% at 3 months. For the numbers and proportion of patients that developed high‐avidity antibodies, see Table 2.

**Table 2:** Proportions of patients in the total study population with avidity index ≥60% after the second dose of vaccine

Time point after the second dose of vaccine	Proportions of patients with avidity index ≥60% after the second dose of vaccine
1 month	43/60 (72%)
3 months	38/60 (63%)
6 months	36/46 (78%)

Concerning the questionnaires, all participants answered after the first dose of vaccine (*n* = 61) but four answers were missing after the second dose (*n* = 57). After the first dose, 52% (*n* = 32) reported side effects and 60% (*n* = 34) after the second dose. There was no significant difference in the numbers of patients who reported side effects between the cohorts or between dose 1 and dose 2 (*p* > 0.05). The three most reported side effects after dose 1 were pain at the injection site (43%), fatigue (21%) and malaise (13%) and after dose 2 pain at injection site (53%), malaise (28%) and headache (25%). No allergic reaction was reported. The duration of the symptoms was only 1–2 days for 91% of the participants that reported side effects. After dose 1, 3% (*n* = 2) reported they needed sick leave due to the side effects and after dose 2, 10% (*n* = 6) reported the need of sick leave.

## DISCUSSION

The main finding was that both the levels of anti‐spike IgG and the avidity index were consistently higher in the cohort of patients with previous COVID‐19 infection. Nonetheless, the majority of patients in both cohorts developed high levels of antibodies, of high‐avidity, after two doses of vaccine.

Our results confirm previous reports showing higher antibody levels after the combination of COVID‐19 infection and vaccination [21, 22, 23]. The levels of anti‐spike IgG have been shown to correlate well with the neutralizing capacity, which in turn is an important factor of protective immunity [11, 24, 25]. Higher levels of anti‐spike IgG can be assumed to reflect a more effective vaccination, although no established cut‐off for antibody levels and protection has been reported [26]. Additionally, several studies have demonstrated that a previous COVID‐19 before vaccination results in higher neutralization titres [22, 27, 28, 29]. Reasonably, a repeated exposure to the virus antigen induces this stronger observed immune response. The numerous reports indicating that only one dose of vaccine does not yield high neutralizing titres or high‐avidity antibodies and that higher antibody levels are seen after the second dose, support this conclusion [16, 30, 31, 32]. Over time, anti‐spike IgG levels diminish in both cohorts and the difference in anti‐spike IgG levels between the cohorts also diminishes, as shown in other studies [24, 29, 33, 34]. Since there is no established cut‐off for protective levels, it is not clear how the declining levels of anti‐spike IgG affects the duration of immunity. Reasonably, declining levels of antibodies indicates a decrease in immunity, but the cellular immune response is also an important factor for protective immunity. Therefore, it is difficult to define a level of protection exclusively based on antibody levels [26].

For the avidity index, we could see a significant difference between the cohorts, with higher avidity index in the COVID‐19 cohort at all time points. This is in line with other studies, which also have found higher avidity index after vaccination of convalescent compared to COVID‐19 naïve patients [28, 35]. However, the absolute difference in the avidity index was at most 17%, but there was a substantial individual variation and overlap between the cohorts. Taken together, this difference may reflect the repeated antigen exposure in the COVID‐19 cohort, although it is difficult to conclude whether the difference between the cohorts will have a clinically relevant effect on the protective immunity. Similarly, the increase of the avidity index in the COVID‐19 naïve cohort over time is relatively small (8%) so it may be argued that the difference may have limited clinical relevance, despite being statistically significant. Our results showed no distinct maturation of the avidity index during the 6 months after vaccination, but instead a plateau already from 1 month after the second dose of vaccine, with the majority of the patients having an avidity index >60%. This is in contrast to others who have shown that after vaccination the avidity increases for at least 6 months after the second dose of vaccine [36, 37]. Our discrepant results might be due to study design and methodological differences between studies. Several different protocols for avidity index exist with different chaotropic agents as well as different concentrations of the agent, which makes it difficult to compare the absolute numerical value of the avidity index between studies. Thus, if higher concentrations of urea had been used, it is possible that further maturation may have been observed. Additionally, there are no standardized definition or cut‐off for high‐ and low‐avidity antibodies and the definition will affect the dynamics as well as the proportion of patients that develop high‐avidity antibodies. However, our results are in accordance with Struck et al, who, with the same definition of high‐avidity, also found that the majority of the patients (78%) developed high‐avidity antibodies after the second dose of vaccine [16].

When compared to natural infection, the avidity index after vaccination is clearly higher, independently of which urea concentration used [15, 16, 18, 20]. That demonstrates a better maturation of the avidity after vaccination and higher avidity, at least in relation to infection. Nonetheless, the results with relatively high‐avidity antibodies, without decrease in the avidity index during the first 6 months after the second dose, are in line with other studies and suggests a sufficient selection and maturation of B cells after vaccination [11, 15, 30, 38]. In other viral infections, it has been shown that failure of avidity maturation is associated with a higher risk of reinfection and since the process of avidity maturation is connected to the proliferation and clonal selection of B cells, it may be concluded that high‐avidity antibodies are likely important to achieve protective and long‐lasting immunity [13, 39, 40]. Whether the maturation of high‐avidity antibodies after vaccination against SARS‐CoV‐2 will generate a long‐lasting immunity, despite a decline in the total level of the antibodies already after 3 months, is not yet clear and future research concerning immunity duration after vaccination is needed.

Another aspect to consider for the longevity and protection of the immune response is the emerge of new virus variants. The first vaccines were based on the SARS‐CoV‐2 Wuhan‐Hu‐1 strain, but when new variants, such as Omicron, with numerous mutations in the spike protein, a less pronounced protection from the vaccine can be expected. The role of the avidity index, antibody levels and previous infections for protection against new infections from different variants is more difficult to predetermine and needs further study. This topic is out of the scoop of the present study but several other studies have shown that infection with SARS‐CoV‐2 before vaccination resulted in a broader immune response with higher neutralization titres and avidity index against the omicron variant compared to vaccination of COVID‐19 naïve [19, 27, 28].

The most reported side effects of the vaccination were, as expected, pain at the injection site and malaise, in agreement with other studies which demonstrate mostly mild side effects and low rates of severe adverse events [5, 6, 41]. Also expected, but important to confirm, is that the majority reported short duration of these side effects. There was no difference in the numbers of patients that reported side effects when comparing the first and second dose or between the two cohorts. Interestingly, even though the side effects were mostly mild and rapidly transient, 10% reported a need of sick leave after the second dose. Other studies generally show higher levels of sick leave after the second dose, with a range between 23.3% and 35%, but with the same pattern with more sick leave after the second dose [42, 43, 44]. One explanation of the discrepant results might be that our hospital planned the working schedule in advance and adjusted the quarantine rules for recently vaccinated to minimize the need of sick leave after vaccination. This might not be a priority medical issue but may be something to consider when planning for large scale vaccinations of a working population, especially in the healthcare sector.

A limitation of the study is the composition of the cohorts. Despite the age distribution being wide, there are no patients over 65 years old, only non‐severe cases of COVID‐19 are included and the large majority are women. Since several studies have shown that there are no differences in levels or avidity of anti‐spike IgG between men and women, the results should reflect populations of younger patients with non‐severe COVID‐19 [18, 20, 37]. As elderly are at risk of severe COVID‐19, this highlights the need of further studies in this population. Another limitation is the relatively small numbers of patients. This have been considered when choosing the statistical methods. For the analysis of the side effects of the vaccine, the small cohort and short duration of follow‐up may affect the ability to detect rare side effects, such as myocarditis that has been reported after vaccination with mRNA vaccine [45].

## CONCLUSION

An immune response with high levels of anti‐spike IgG after vaccination was seen in both cohorts and the majority developed high‐avidity antibodies, although both antibody levels and avidity index were higher in patients with previous COVID‐19 infection. Over time, the levels of anti‐spike IgG declined in both cohorts, but a high‐avidity index prevailed. Vaccine side effects were mild and of short duration but up to 10% needed sick leave.

## FUNDING INFORMATION

This work was supported by The Region Halland Research council, the Foundation of Sparbanken Varberg and the Southern Medical Care Region, Sweden.

## CONFLICT OF INTEREST STATEMENT

The authors declare no conflicts of interest. The authors declare that they have no known competing financial interests or personal relationships that could have appeared to influence the work reported in this paper.

## Data Availability

The data that support the findings of this study are available from the corresponding author upon reasonable request.
